# Senolytic vaccination: a new mandate for cardiovascular health?

**DOI:** 10.20517/jca.2022.03

**Published:** 2022-03-11

**Authors:** Travis B. Lear, Toren Finkel

**Affiliations:** 1 Aging Institute, University of Pittsburgh/UPMC, Pittsburgh, PA 15219, USA.; 2 Vascular Medicine Institute, University of Pittsburgh, Pittsburgh, PA 15213, USA.

**Keywords:** Senolytic vaccination, aging, GPNMB

## Abstract

Senescent cell accumulation is increasingly associated with a number of age-related cardiovascular diseases. Now, a new manuscript in *Nature Aging* suggests that a novel vaccine-based strategy might provide a targeted method to eliminate the senescent cell population.

Aging occurs at both the organismal and cellular levels. For the latter case, cellular aging is often viewed synonymously with cellular senescence, a cell fate change characterized by permanent cell cycle arrest. Now, a new study published in *Nature Aging* details a therapeutic strategy that seeks to eliminate senescent cells through vaccination. Current thinking often views senescence both as a protective and potentially harmful phenomenon. Following non-lethal DNA damage, entry into senescence is viewed as an evolutionary protective strategy, wherein the now non-dividing senescent cell is prevented from undergoing malignant transformation. However, increasingly, the accumulation of senescent cells has been linked to numerous diseases of aging. Despite their growth arrest, senescent cells continue to influence their surroundings, and can produce and secrete a panoply of pro-inflammatory cytokines and signals. Together, this behavior is known as a *Senescence-Associated Secretory Phenotype* (*SASP*), leading to a form of sterile inflammation which can cause local tissue remodeling and widespread systemic effects. Over the past decades, senescent cells have been shown to accumulate across numerous aged tissue and cell types. However, senescent cells are increasingly appreciated not only as markers for aging but as drivers of disease, including cardiovascular diseases^[1]^. For instance, senescent cell number has been noted to increase greatly in the aged vasculature^[2]^. A study of atherosclerosis in both animal models and patient samples revealed that senescent vascular foam cells contribute to multiple stages of plaque progression^[3]^. Furthermore, in a high-fat diet model, mice strains prone to develop early senescence also exhibited increased endothelial dysfunction and an accelerated onset of heart failure^[4]^.

A challenge to understanding senescence has been the lack of a consensus for what exactly constitutes a senescent cell. Consistent with growth arrest as a primary manifestation, an increase in inhibitors of the cell cycle pathway often form the primary markers of cellular senescence; this includes the cyclin-dependent kinase inhibitors p16^Ink4a^ and p19^arf^, and the tumor suppressor p53. Other markers used to identify senescent cells include telomere length, the appearance of DNA damage markers, and increased activity of senescence-associated β-galactosidase (SA-β-gal). Utilizing a constellation of these types of molecular markers, several groups have demonstrated that removing senescent cells can have important biological effects. In a specially designed mouse model, selective removal of p16^Ink4a^ positive cells appeared to delay age-related pathologies^[5]^. Spurred by promising results from these specialized genetic models, significant effort has been directed at developing pharmacological tools to enhance the clearance of senescent cells, termed *senolytic agents*. Current agents include the combination of the tyrosine kinase inhibitor dasatinib (D) and the flavanol quercetin (Q)^[6]^. This combination (*D* + *Q*) has already been tested in human clinical trials for patients with idiopathic pulmonary fibrosis^[7]^, a disease in its inherited form characterized by defects in telomere maintenance. Other agents such as ABT-263 (Navitoclax) are also being developed^[8]^. While promising, the current state of senolytic therapeutics is nascent, and current agents suffer from toxicity issues^[9]^, suggesting the need for additional approaches.

In a recent study in *Nature Aging*, Suda *et al.*^[10]^ suggested that rather than using small molecules, harnessing the power of the immune system might be the key to ridding the body of senescent cells [Figure 1]. In particular, this group used a vaccine-based approach to selectively eliminate senescent cells. Although a form of this approach has been described previously^[11]^, identification of a truly senescence-specific antigen has remained a significant obstacle. To overcome this barrier, Suda *et al.*^[10]^ first analyzed transcriptome datasets from senescent cells to identify genes associated with senescence. They further limited their analysis to those genes with predicted transmembrane regions - key for identifying extracellular-facing regions to act as putative senescence-specific antigens. From these curated hits, they identified the transmembrane protein *glycoprotein nonmetastatic melanoma protein B* (GPNMB). The authors next confirmed that GPNMB expression increased on senescent vascular endothelial cells and in atherosclerotic patient samples. The researchers next generated a transgenic mouse with a luciferase reporter under the control of the *Gpnmb* promoter. Following 12 weeks of a high-fat diet, mice exhibited an increased luminescent signal consistent with increased GPNMB expression. In addition to the luciferase reporter, they also introduced a human diphtheria toxin receptor into the *Gpnmb* locus. In these animals, treatment with diphtheria toxin leads to the selective elimination of GPNMB-expressing cells. In mice fed a high-fat diet, diphtheria toxin led to reduced *Gpnmb*-driven luminescence, as well as a reduction in senescent cell accumulation. Further, elimination of these GPNMB-positive cells ameliorated the metabolic dysfunction seen in these high-fat fed mice. The group then extended these studies to an atherosclerosis mouse model, where elimination of GPNMB-positive cells in an Apolipoprotein E KO mouse (ApoE KO) model also decreased the aortic plaque area.

Having identified GPNMB as a possible senescence-specific antigen, the group next designed peptides corresponding to the extracellular domain of the protein to initiate a vaccination-based strategy to target GPNMB positive cells. They confirmed that in mice, administration of this GPNMB-based vaccine elicited an antibody-dependent cellular cytotoxic response mediated by natural killer cells, which could dose-dependently eliminate GPNMB-expressing cells. With this tool in hand, the group explored the potential of their GPNMB vaccine. Remarkably, mice fed a high-fat diet for 12 weeks and immunized with the GPNMB vaccine 4 weeks into treatment displayed decreased GPNMB expression, reduced senescent cell burden, and improved metabolic parameters. Importantly, the GPNMB-based vaccine also reduced atherosclerotic plaque burden in the ApoE KO mouse model. Given the potential connection between senescence and aging itself, the group next sought to test this approach in a mouse model of Hutchinson-Gilford progeria syndrome, an accelerated aging model. Interestingly, they observed that GPNMB vaccination prolonged median lifespan from 21 to 25 weeks (20%) in this accelerated aging model characterized by vascular aging. Finally, Suda *et al.*^[10]^ tested the efficacy of GPNMB vaccination against other current senolytic therapies, including *D* + *Q* and Navitoclax. In the high-fat diet model, vaccination appears to show comparable effects in reducing senescence cell burden after 16 weeks and appeared superior to current small molecule approaches at a 24-week time point. As such, this study demonstrates that the vaccine-mediated elimination of senescent cells holds promise to treat cardio-metabolic conditions, as well as other vascular and age-related phenotypes.

The target of these vaccination efforts, GPNMB, has been the focus of several previous research studies. Originally identified as a glycoprotein in melanoma cell lines, GPNMB is now recognized to be widely expressed across tissue and cell types and plays multi-faceted roles in inflammation and disease^[12]^. Moreover, a soluble fragment of GPNMB can be released following cleavage by the metalloprotease ADAM10, and this fragment has been observed to enhance endothelial migration *in vitro*^[13]^. At present, it is unclear if cleavage of GPNMB or fragment activity is associated with senescence. Intriguingly, GPNMB was also identified as a potential secreted biomarker following activation of a key regulator of cellular energetics, AMP-activated protein kinase (AMPK)^[14]^. AMPK functions as a key metabolic switch by sensing the ratio of AMP to ATP and is activated in the setting of energetic stress. Given the results of Suda *et al.*^[10]^, it will be interesting to see whether there is a connection between AMPK activation and GPNMB expression in senescent cells.

Finally, the results from Suda *et al.*^[10]^ add to the ongoing debate as to the full biological implications of senescent cells. Numerous studies have attested to the deleterious effect of senescence cells; however, recent results suggest a more nuanced view. For instance, Grosse *et al.*^[15]^ observed that hepatic endothelial cells in mice aged 12 months displayed high p16^Ink4a^ expression. Rather than improving the phenotype, elimination of these p16^Ink4a^ expressing cells resulted in liver fibrosis and disease. Indeed, the role of GPNMB itself remains unresolved. Recently published studies demonstrated that in young mice, GPNMB might play a protective role in metabolic disorders, with Gpnmb KO mice apparently showing impaired metabolic parameters and heightened inflammation stemming from macrophage activation^[16,17]^. Besides delineating the beneficial or harmful role of GPNMB, how this protein intersects with other markers of senescent cells, such as p16^Ink4a^, p19^arf^, and p53, remains unresolved. Are all p16^Ink4a^-high senescent cells likewise GPNMB-high, and are all GPNMB-high cells truly senescent? Moreover, could GPNMB expression denote a more deleterious subtype of senescent cells, opening the potential for more specific targeting of good versus bad senescent cells? The answers to these questions and others must await additional analysis. What’s clear is that harnessing the immune system represents a new and potentially powerful strategy to clear senescent cells and potentially treat a host of age-related cardiovascular diseases.

## Figures and Tables

**Figure 1.**
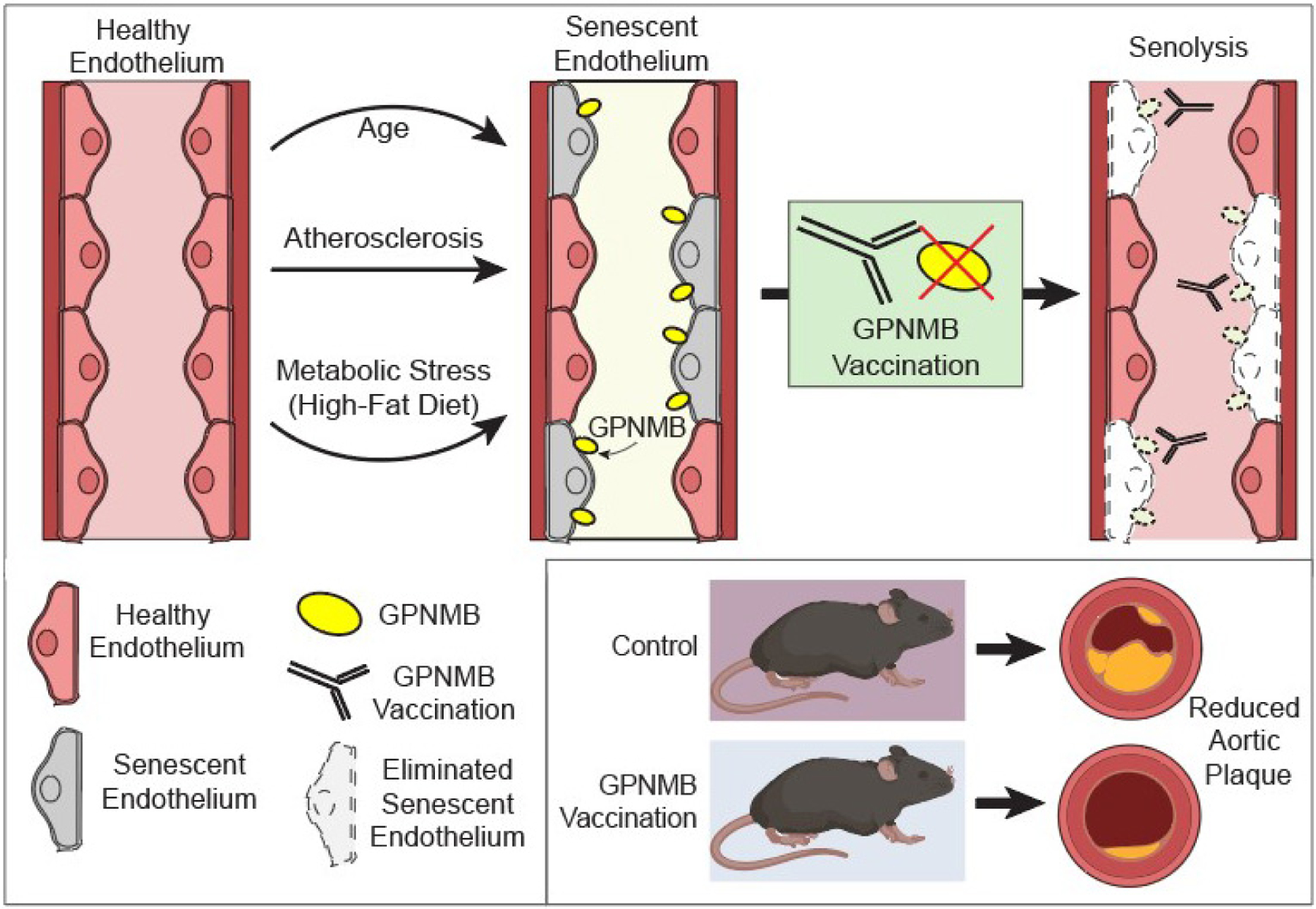
*Glycoprotein nonmetastatic melanoma protein B* (GPNMB) as a vaccine target for senolysis. Suda *et al.*^[10]^ utilized RNAseq data on senescent cells to uncover GPNMB as a transmembrane protein disproportionately upregulated in senescent endothelial cells. GPNMB-based vaccination protected mice against vascular plaque burden, and extended lifespan in an accelerated aging model characterized by vascular pathology. Pink cells represent healthy cells; grey cells represent senescent cells; yellow icons represent GPNMB protein that accumulates on aged and senescent endothelial cells; white outlined cells represent eliminated senescent cells from senolytic vaccination.
